# Supportive care: men’s expectations who undergoing in vitro fertilization treatment

**DOI:** 10.1186/s13104-020-05407-5

**Published:** 2020-12-07

**Authors:** Roghieh Bayrami, Roksana Janghorban, Fatemeh Effati-Daryani, Masoumeh Hajshafiha

**Affiliations:** 1 Department of Midwifery, Reproductive Health Research Center, School of Nursing and Midwifery, Urmia University of Medical Sciences, Urmia, Iran; 2 Department of Midwifery, School of Nursing and Midwifery, Community Based Psychiatric Care Research Center, Shiraz University of Medical Sciences, 7193613119 Shiraz, Iran; 3 Department of Midwifery, School of Nursing and Midwifery, Urmia University of Medical Sciences, Urmia, Iran; 4 Department of Obstetrics and Gynecology, School of Medicine, Reproductive Health Research Center, Urmia University of Medical Sciences, Urmia, Iran

**Keywords:** Counseling, Fertilization in vitro, Infertility, Qualitative research, Reproduction

## Abstract

**Objective:**

Infertile couples perceive some needs and expectations during treatment. Most studies have focused on infertile women’s needs. The study was done to explore the expectations of men who undergoing in in vitro fertilization (IVF) treatment. Participants were men whose wives undergo IVF treatment in the IVF clinic of Shahid Motahhri hospital in Urmia. Purposive sampling was performed and data collection was done through in-depth semi-structured interviews until data saturation.

**Results:**

“Supportive care” was emerged as the main theme consisted of two categories. The first category was “to be treated with attention in healthcare setting” which included three subcategories: Receiving couple based care, establishing counseling centers, and continuous care. The second category was “to be welcomed in the society” which included two subcategories: Financial support from community and close family, and changing community views about IVF treatment. Expectations of men undergoing IVF focuses on supportive care. They seek to receive the kind of care from health care setting and society. A deep understanding of the needs could help practitioners to consider men’s expectations, and assist policy makers and researchers to design and implement interventions and programs in infertility clinics which enhance the partnership of infertile men and fulfill their expectations.

## Introduction

Infertility is a life crisis and infertile couples perceive some needs and expectations during treatment [1–3]. Global pattern of infertility showed that absolute number of infertile couples has increased and over 52 million couples in 2010 [4]. Infertility rate trend has been increased from 2.8% in 2001 to 24.9% in 2010 in Iranian couples [5]. Therefore, infertility treatment has become increasingly and women are the center of most infertility treatments in the increased rate [6]. Remarkable studies showed that women undergoing in vitro fertilization (IVF) experienced anxiety, depressive symptoms, and poor quality of life [7, 8]. In spite of gender differences in psychological and emotional reactions during diagnosis and treatment of infertility, it affects men in various situations [2, 9]. They could be as a partner of an infertile woman or they could encounter with their own infertility or they could experience unexplained infertility as an infertile couple [10, 11].

The psychological symptoms are increased during diagnosis and treatment of infertility or even the transition to fatherhood in men with infertility history [12, 13]. Since infertility is a multifaceted problem, its effects could not limit to emotional consequences [14]. To our knowledge, most studies have focused on male psychological well-being and needs in infertility but less is known about the multi-dimensional needs of infertile men seeking assisted reproduction treatment (ART) and their thoughts and experiences regarding their fertility care [15–17]. There is also evidence that men, unlike women, rarely express their problems and needs in the health service [18, 19]. Considering to existing gap, the qualitative research was done to explore the expectations of men who undergoing in IVF treatment.

## Main text

### Methods

A qualitative study was selected for this study. This approach is appropriate when previous studies have not focused on understudied subject and deep descriptions of a phenomenon are desired [20]. The study was conducted in the IVF clinic of Shahid Motahhri educational hospital in Urmia, which is referral center for infertility couples assessment in West Azerbaijan Province of Iran. 

#### Inclusion criteria

Men who experienced IVF treatment for their wives were included in this study. 28 individual interviews with 26 participants (19 men, 2 gynecologists, 3 wives, 2 midwives) were performed. Inclusion criteria of men's participants were Iranian, speaking in Persian language, fertile or infertile men was monogamous whose wives undergoing IVF with own sperm and ovum, not having step-child and the tendency to share their experiences. Exclusion criteria were having history of divorce and remarriage, and gamete donation.

#### The data collection tools

Data were collected through in-depth, semi-structured, face-to-face interviews using interview guide which has previously been published [21]. Interviews lasted 45–100 min. Interviews were conducted in a private room in the hospital to assure their confidentiality and comfort. A second round of interviews was also conducted for two participants. The interviews were voice recorded and immediately transcribed verbatim.

#### Setting and recruitment

In the present study, purposive sampling was performed. In order to increase exploring different opinions in this group, maximum diversity of men's characteristics including age, educational level, employment, cause and duration of infertility was considered.

#### Analysis

Data were analyzed using Graneheim and Lundman qualitative content analysis method [22]. Similar codes were classified into subcategories and then were made into a category. Meanings was extracted in categories emerged a comprehensive meaning as the main theme. Data analysis was conducted by MAXqda software version 10. The study was approved by the Research Ethics Committee of Urmia University of Medical Sciences (IR.UMSU.REC.1396.127). Written informed consent was signed by all participants before each interview. Confidentiality was ensured at all stages of the research. The trustworthiness of the research was considered according to Lincoln and Guba's measures [23].

### Results

“Supportive care” was the men’s expectation who their wives undergoing IVF treatment that emerged as the main theme. This theme consisted of two categories: to be treated with attention in healthcare setting with three subcategories, and to be welcomed in the society with two subcategories (Table 1).

**Table 1:** Subcategories, categories, and theme emerged from the study

Subcategory	Category	Theme
Receiving couple based care Establishing counseling centers Continuous care	To be treated with attention in healthcare setting	Supportive care
Financial support from community and close family Changing community views about IVF treatment	To be welcomed in the society	

#### To be treated with attention in healthcare setting

##### Receiving couple based care

Men needed “receiving couple- based care” in all procedures, treatments and intervention. Men expressed that they expected involvement in the treatment both as a man and as a couple. They did not wish only to be place on the side-line. One man commented, “*They should allow me to decide on whether to planned sexual intercourse or to start IVF process*”. [Man 12, 30 years old, 5 years infertility, female cause].

Men did not have enough participation in planning for pregnancy. One participant stated: “*Unfortunately, in our society only women are considered in infertility care and men is neglected or ignored*”. [Man 2, 31 years old, 4 years infertility, female cause].

One of the midwives stated: “*The health of couples undergoing IVF treatment affects the child’s health. We do not pay attention to the issue, and we believe that only mothers should be cared for*”. [Female midwife, 32 years old, 8 years of work experience].

##### Establishing counseling centers

Participants voiced a variety of preferences for receiving support, and counseling from IVF clinic. They believed that couples’ involvement in counseling sessions is essential. Participants pointed out that there is nowhere to address their physical, sexual and psychological concerns before and after the IVF treatment. One gynecologist said:

“*Unfortunately women rarely revisit us for their sexual concerns after the IVF but I think the most suitable person to help them is a sex therapist in a consultation center. Additionally their spouses' needs should be considered alongside them*”. [Female gynecologist, 52 years old, 20 years of work experience].

Another participant implied, “*The doctors or other providers at the IVF clinic do not provide the psychological counseling and support that we need. I hope that there is a psychological counseling center that can provide us support*”. [Man 9, 32 years old, 7 years infertility, female cause].

##### Continuous care

Some participants wanted continuous care. They liked to doctors and health care providers work within team. One participant said, “*To maintain a care chain it would help if doctors would discuss treatment possibilities together, adjust one order and write this down in the patients’ record and then he/she implements the treatment*”. [Man 18, 34 years old, 5 years infertility, female cause].

The majority of men believed that care from preconception period until IVF treatment and after that time should be provided by a certain team. On the other hand paying attention to the transition of patients and documents between clinics in different cities is important because it interrupts continuous care. One of midwives said, “*Cares must be taken in a team from before to after of IVF. It might be necessary patients can be followed-up*”. [Female midwife, 45 years old, 15 years of work experience].

#### To be welcomed in the society

##### Financial support from community and close family

Difficulty in finding drugs, long distance to the IVF treatment center and financial problems were some of the difficulties that many participants encountered with it.

One of participants said, “*Only I am employed and I lose all income on the days that we attended clinics*”. [Man 13, 33 years old, 6 years infertility, female cause]. Another man implied, “*We had borrowed a loan from the bank and spent it for the treatment*”. [Man 17, 39 years old, 7 years infertility, female cause].

Most of participants came from other cities. The participants expected that their near family and friends would be support to assist them. One participant said, “*The IVF treatment center is in Urmia. We had to stay in the hotel at night. We have near family and friends in Urmia and we expect to support and hold our hands in this city*”. [Man 5, 36 years old, 9 years infertility, male cause].

Majority of participants believed that the insurance services are needed to help more. One of the participants said, “*Buying the drugs was difficult and the insurance services did not help us*”. [Man 8, 32 years old, 4 years infertility, female cause].

##### Changing community views about IVF treatment

Some participants wanted the community's positive attitude towards male infertility and IVF treatment. Majority of men felt a sense of stigma while engaging in treatments which in conflict with their masculine sense. One man said: “*I am afraid that around people think I cannot produce sperm. This is a taboo in our community and it was a reason that I wouldn’t want to tell someone about IVF treatment*”. [Man 8, 30 years old, 6 years infertility, female cause].

### Discussion

Iranian men who their wives undergoing IVF treatment need to “supportive care” as the main expectation. Men expected to receive couple- based care and did not want only to be placed on the side-line. The present findings agree with the results obtained in Arya and Dibb study. They showed that men reported feeling dismissed from the treatment process and their contribution was ignored [24]. In the response of the necessity of the couple-based care, Ying et al. developed the partnership and coping enhancement for couples undergoing IVF treatment [8].

Participants expected to access to counseling center. They sought to take advice from professionals about their sexual concerns and psychological distress. The need was not met due to lack of enough time of medical professional during treatment process. The findings confirmed by Roudsari and Allan which argued professionals must implement holistic approach in infertile couple counseling for fulfillment of their multifaceted needs in psychological, social and cultural issues [14]. Attention to psychological and emotional needs is very essential especially in Iranian infertile couples because prevalence of depression in these couples was high [25]. Besides, previous studies showed that men's depressive symptoms increase in male factor infertility and more attention to their emotional counseling is necessary [26, 27]. Although the cause of infertility in half of participants in the current research was different from the cited studies, their remarks showed that establishing counseling center can meet their various needs.

Continuous care was the other men's expectations. They preferred a certain team was responsible of their treatment process. The present findings agree with the results of previous studies showed that continuity is a dimension of patient centered care which was considered very little and were related to higher tolerability of treatment and lower depression [28–30].

Participants needed to financial support from community and close family. They endure many problems due to experience IVF failure, repeated IVF cycles, and lack of insurance coverage of infertility treatment. These financial problems increased when they have to go to another city for treatment. The present findings concur with the results of Hossein Rashidi et al. study that 61% of couples had economic difficulties and inaccessibility of insurance coverage for infertility services was the most important cause of treatment discontinuation [31]. The result was confirmed by a study showed that patients' out- of- pocket payment for infertility treatment is “considerable expenditure” in developing countries [32].

Men expected the community's positive attitude towards infertility. They did not tell about their infertility, treatment process to close family and other friends due to fear of stigma around the infertility in the society. They felt a deficit which was in conflict with the definition of their masculinity. Some studies showed that Iranian infertile couples experience the stigma and tendency to concealing infertility and doing IVF as a treatment method from their relatives [33, 34]. The results of Arya and Dibb's study agree with the current study that showed that perceived stigma and unsupported family is men's experiences during infertility treatment [24]. A study showed that decision to secrecy intensifies in donor insemination, egg donation and surrogacy [35]. Although, IVF was done using couples' sperms and ovums, in the current study, but results showed that men was stigmatized and had a tendency to hide. In conclusion, this study showed that expectations of men undergoing IVF focuses on supportive care. They seek to receive the kind of care from health care setting and society. A deep understanding of the needs could help policy makers and health professionals to design and implement interventions which enhance the partnership of infertile men and fulfill their expectations.

## Limitations

The present findings are also limited by the issue that only male or female factor were found as a cause of participants' infertility. Unexplained cause of infertility may influence men's expectation.

## Data Availability

The dataset supporting the conclusions of this article is included within the article.

## Abbreviations

IVF: In vitro fertilization
MAXQDA: MAX Qualitative Data Analysis
ART: Assisted reproductive technology
